# Long-Term Trends in Water Quality Indices in the Lower Danube and Tributaries in Romania (1996–2017)

**DOI:** 10.3390/ijerph18041665

**Published:** 2021-02-09

**Authors:** Rodica-Mihaela Frîncu

**Affiliations:** 1 National Institute for Research and Development in Chemistry and Petrochemistry—ICECHIM, 202 Splaiul Independentei, 060021 Bucharest, Romania; icechim.calarasi@gmail.com; Tel.: +40-21-315-3299; 2 INCDCP ICECHIM Calarasi Branch, 2A Ion Luca Caragiale St., 910060 Calarasi, Romania

**Keywords:** Danube, water quality index (WQI), Romania, principal component analysis (PCA), nutrients pollution, long-term monitoring, municipal wastewater, sewerage pollution

## Abstract

The Danube River is the second longest in Europe and its water quality is important for the communities relying on it, but also for supporting biodiversity in the Danube Delta Biosphere Reserve, a site with high ecological value. This paper presents a methodology for assessing water quality and long-term trends based on water quality indices (WQI), calculated using the weighted arithmetic method, for 15 monitoring stations in the Lower Danube and Danube tributaries in Romania, based on annual means of 10 parameters for the period 1996–2017. A trend analysis is carried out to see how WQIs evolved during the studied period at each station. Principal component analysis (PCA) is applied on sub-indices to highlight which parameters have the highest contributions to WQI values, and to identify correlations between parameters. Factor analysis is used to highlight differences between locations. The results show that water quality has improved significantly at most stations during the studied period, but pollution is higher in some Romanian tributaries than in the Danube. The parameters with the highest contribution to WQI are ammonium and total phosphorus, suggesting the need to continue improving wastewater treatment in the studied area. The methodology and the results of the study may be very useful instruments for specialists and decision makers in updating river basin management plans and prioritising intervention measures.

## 1. Introduction

The Water Framework Directive (WFD) [1] of the European Union (EU) has the aim to achieve good ecological status in all EU water bodies and to protect them from pollution, which affects aquatic ecosystems and human health. It was adopted in year 2000 and it introduced a holistic approach in the management of water resources, based on river basin management plans [2].

Surface waters provide important ecosystem services, including drinking and irrigation water, hydropower, navigation etc. Contamination of water bodies intended for abstraction of drinking water may pose significant public health risks, leading to infectious diseases [3,4,5], while the ingestion of nitrates leads to methemoglobinemia, blocking oxygen transport to the cells, particularly in infants [6]. Recent studies investigate the link between nitrates and reproductive and developmental disorders [7], while nitrites may be linked with increased risk of childhood brain tumors [8] and different types of cancer in adults [6].

Water pollution may originate in point sources (municipal and industrial discharges of treated or untreated wastewater) or diffuse sources, like run-off from agricultural land [9]. After treatment in the drinking water supply facilities, tap water may be contaminated in the distribution network with algal toxins and heavy metals from pipes corrosion, which are directly linked with a wide range of diseases. Moreover, some of the products used for water disinfection may have negative health impacts if not removed [10]. This has lead in many regions of Europe to distrust in drinking tap water and a preference for bottled water [5].

In the European Union (EU), Council Directive 98/83/EC, also known as the Drinking Water Directive (DWD), has the purpose of ensuring that safe water is provided to citizens in order to protect public health [11]. In 2020, the EU Council has approved a proposal to revise the directive, updating quality standards and including endocrine disruptors and pharmaceuticals in the monitoring programme [12]. Several countries have applied for derogations from the DWD, including Italy, Romania, and Hungary [13,14]. According to the WFD, water quality assessment includes chemical status and ecological status of water bodies based on parameters that are monitored on a regular basis by national authorities and reported to the EU.

The Danube River is the second longest in Europe and its catchment area includes 19 countries, making it the most international river in the world [15]. Millions of people are relying on the Danube as drinking water source [16,17] and its contamination can have serious impacts on human health [18,19].

Monitoring data is used to assess water quality and identify pollution sources, so that river management plans can include the most appropriate measures to control and reduce pollution, and to ensure sustainable water use. The progress towards the goals and the effectiveness of the measures are assessed by the evolution of water quality in time. In this respect, nutrients were for a long period the main focus of monitoring and research [20], particularly because municipal wastewater treatment was non-compliant in many areas of the Danube Basin [21,22]. Advanced scientific programmes with international teams of experts were dedicated to modelling nutrient discharges in the Danube and international agreements and regulations were adopted to reduce nutrient pollution [23,24,25,26]. In recent years, a much stronger focus is on ecological assessment [27,28] and on emerging micropollutants, like pharmaceuticals and endocrine disruptors, which may have a strong environmental impact even in small amounts [29].

There are several scientific studies that analyzed individual parameters in the Danube and how they evolved over longer periods of time [30,31] and a number of studies that looked at the complex interaction between parameters [32] and between parameters and their drivers [33,34,35,36,37,38,39]. However, to support authorities and decision makers, and to communicate information to the public, an overall assessment of water quality can be a very useful tool.

Water quality indices (WQI) have been developed in order to assess the adequacy of using the water for specific purposes, by aggregating several monitored parameters into a single number that can be used by specialists and authorities to define water policy and prioritize management measures. WQI is one of the 25 indicators that can be used to assess the environment, called environmental quality indices, EQI [40]. WQI calculations are based on monitoring data of selected parameters and weights that often require expert opinion. Thorough reviews of existing methods and their limitations have already proved that applying different methods on the same dataset may lead to different results [41,42,43]. A possible solution in this case, which has the advantage that it does not need expert judgement for ranking parameters, is to calculate WQI based on limit values from national water quality standards [44,45]. The weighted arithmetic method used in this study is based on the National Sanitation Foundation-Water Quality Index and has already been applied with good results in different river basins [44,46], as well as for the Danube in Romania in the area of Galati for a period of 4 years [17].

When assessing long-term trends in water quality, the most important challenge is the availability of consistent monitoring data for the studied area [47,48]. In this respect, the Third Joint Danube Survey, organized in 2013 by the International Commission for the Protection of the Danube River (ICPDR), has found chemical monitoring data to be consistent and reliable throughout the Danube River Basin, while ecological monitoring methodologies still need to be harmonized and further developed [15].

The present paper is part of a larger study on long-term trends in main water quality parameters in the Lower Danube [49]. The parameters belong to the core physical and chemical monitoring plans and were selected according to data availability at the studied sites. In the first phase, each parameter was analyzed individually to see how it evolved during the period 1996–2017 [50]. However, not all parameters are equally important for ecosystems balance, so, in order to assess the overall water quality and long-term trends, it is useful to calculate an index that includes several parameters and gives them corresponding weights. WQI was calculated using the weighted arithmetic method for 15 locations in the Lower Danube and Romanian tributaries based on annual means of 10 parameters for a period of 22 years (1996–2017), and their trends in time were assessed using Spearman rank correlation. Principal component analysis (PCA) indicates parameters’ contributions to the index and correlations between them, while factor analysis highlights differences between locations. The present paper shows how WQI reflects chemical water quality in the Lower Danube and tributaries during the period 1996–2017 and is a step towards modelling future trends.

The main aims of the study are:

- to assess water quality in the Lower Danube and 6 Romanian tributaries during the period 1996–2017, based on WQI, using 10 core physical-chemical parameters;
- to analyze long-term water quality trends during the study period at the selected locations;
- to identify correlations between parameters and compare water quality at different locations using advanced statistical methods.

To the best of the author’s knowledge, this is the first study to analyze WQIs in the Lower Danube and Romanian tributaries for a long period of time.

## 2. Materials and Methods

### 2.1. Study Area

The Danube River originates in the Black Forest Mountains, in Germany, and flows through 10 European countries before draining into the Black Sea. It is the second longest river in Europe, after the Volga, and has a high social and economic importance, serving as drinking water source, navigation way, hydropower generator, and performing a large number of ecosystem services, including provisioning, regulating and cultural services [51]. 97.4% of Romania’s territory is located in the Danube basin, representing 29% of its catchment area. Efforts to monitor and reduce pollution are coordinated by the ICPDR, located in Vienna [21]. In this respect, the TransNational Monitoring Network (TNMN) was established in 1996, and currently national authorities in charge of monitoring water quality in the Danube basin submit their data to the ICPDR to be included in its database [52]. The TNMN was revised in 2007 in order to meet the requirements of the WFD [21].

National authorities also support their own research, so in Romania several studies were published related to this topic [20,53,54].

**Figure 1:**
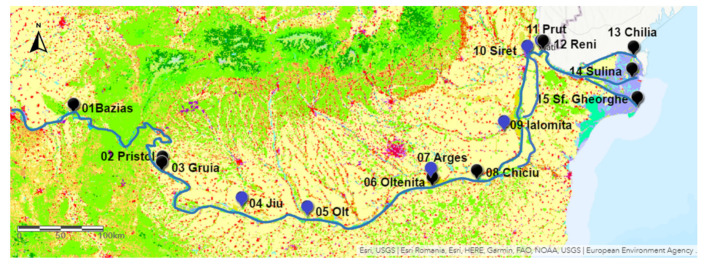
CORINE land cover map of the study area in the Lower Danube with representation of the sampling points (01–15), Danube—black, tributaries—blue [55].

The study area is in the Southern part of Romania and includes 9 monitoring stations located on the Danube, from Bazias to the Black Sea (1071 km), and 6 monitoring stations located on the Romanian tributaries Jiu, Olt, Arges, Ialomita, Siret, and Prut, close to their mouths, where they drain into the Danube. The locations of the monitoring stations are presented in Figure 1, and more details regarding their geographic coordinates, position along the river, size of catchment area and discharge are included in Table 1.

The CORINE land cover map in Figure 1 shows that the study area consists mainly of plain non-irrigated agricultural land in the South, and forests in the mountain areas in the North. The map legend is included in the Supplementary Materials (S3).

The selected monitoring stations are part of the TNMN. The study includes only stations that are monitored by the National Administration “Romanian Waters”, who reports data to the ICPDR. Sampling and analysis of water samples is carried out according to international standards, by national River Basin Management authorities. For some stations monitoring data are available for the period 1996–2017, the others were obtained for the period 2007–2017 (Gruia, Jiu, Olt, Ialomita).

### 2.2. Data

Monitoring data were retrieved from the ICPDR Water Quality Database [52], with the permission to use them for scientific studies. The analyzed parameters are pH, dissolved oxygen (DO), biochemical oxygen demand (BOD_5_), chemical oxygen demand (COD-Cr), ammonium nitrogen (NH_4_^+^-N), nitrates nitrogen (NO_3_^−^-N), total phosphorus (TP), total suspended solids (TS), chlorides (Cl^−^), and sulfates (SO_4_^2−^). The parameters were selected as most commonly used and relevant for chemical assessment of water quality, according to literature [41,42,45], depending also on data availability.

For some of the stations, the database includes values from three river sections: left bank (L), middle (M), and right bank (R). In this study only data from the middle point (M) were analyzed, because they were available for all stations. Only monitoring stations operated by Romanian authorities were included in this study.

**Figure 2:**
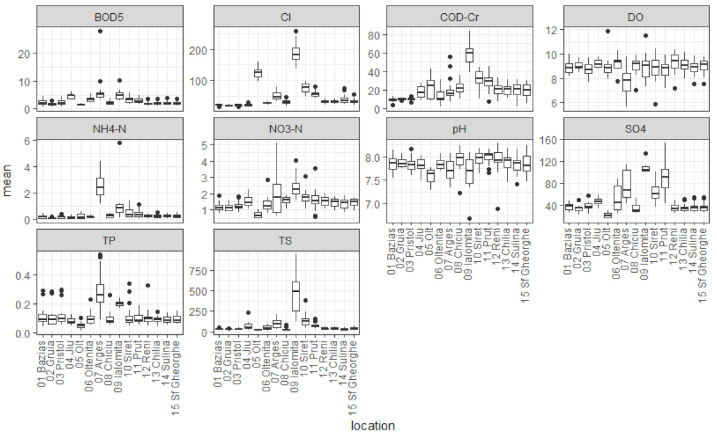
Boxplot representation of mean annual values for analyzed parameters at each sampling location during the period 1996–2017. Parameters are BOD_5_ (mg O_2_/L), Cl^−^ (mg/L), COD-Cr (mg O_2_/L), DO (mg O_2_/L), NH_4_^+^-N (mg N/L), NO_3_^−^-N (mg N/L), pH, SO_4_^2−^ (mg/L), TP (mg P/L), TS (mg/L).

Data were downloaded into an excel table and were further processed using Microsoft Excel and the open source R Statistical Software, version 3.6.2. Annual means were calculated for each parameter at each location. The number of observations for each location are presented in Table 2, and the annual mean values are presented in Figure 2.

### 2.3. Water Quality Index Method

The purpose of the study was to calculate a water quality index taking into account a series of physical–chemical parameters that are relevant for an overall assessment of Danube water during the period 1996–2017, in order to compare the analyzed stations and see how quality has evolved during this period.

Water quality indices (WQI) were calculated using the weighted arithmetic water quality Index method, with Equation (1) [42]:

(1) $$\mathrm{WQI}=\;\frac{\sum Q_{i}\cdot W_{i}}{\sum W_{i}}$$

where Q_i_ is the quality rating scale of parameter i and W_i_ is the weight corresponding to parameter i, calculated using Equations (2) and (3), respectively.

(2) $$Q_{i}=100\cdot \;\frac{V_{i}-V_{0}}{S_{i}-V_{0}}$$

(3) $$W_{i}=\frac{K}{S_{i}}$$

V_i_ is the annual mean of parameter i, V_0_ is the ideal value of the parameter and S_i_ is the standard limit value for parameter i. V_0_ = 0 for all parameters, except pH, for which the ideal value is 7 and DO with ideal value 14.6 mg O_2_/L. S_i_ values are Romanian standard values for quality class II [58], because the Danube water quality is generally considered to belong to this class [17]. K is a proportionality constant, calculated using Equation (4):

(4) $$K=\;\frac{1}{\sum \left(\frac{1}{S_{i}}\right)}$$

The limit values S_i_ and the calculated weights W_i_ are presented in Table 3.

According to this method, water quality is assessed by WQI values as seen in Table 4.

### 2.4. Statistical Analysis

Trend analysis at each monitoring location was carried out by calculating Spearman rank correlation coefficients, ρ, between WQI values and the year for which they were calculated.

Principal component analysis (PCA) was applied on sub-indices (Qi·Wi/$\sum W_{i}$) with the aim of finding out which parameters have the strongest influence on the WQI and how parameters are correlated. Because parameters usually have different units of measure and orders of magnitude, data are scaled and centered before analysis to avoid wrong interpretation. In this case, parameters are scaled during WQI calculation, but then are weighed, so PCA was carried out with and without centering and scaling data, to compare both situations.

Factor analysis is a statistical method that can be applied on datasets that contain both numerical and categorical variables or factors. It was applied on sub-indices according to their locations in order to highlight the differences between the monitoring stations.

All results and graphics were generated using Microsoft Excel and the open source R Statistical Software, version 3.6.2. and RStudio Version 1.2.5033.

## 3. Results

### 3.1. Water Quality Index

**Figure 3:**
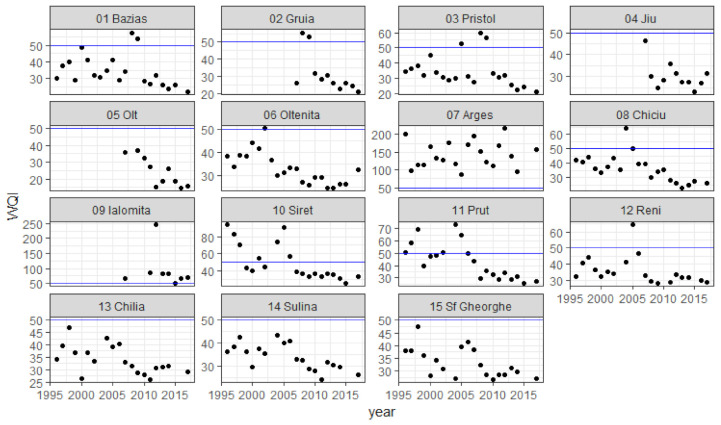
Scatterplot of WQI values at each location during the period 1996–2017 (blue line = limit between “good” and “poor” water quality, WQI = 50).

The calculated WQIs for each location and year are included in Table 5 and represented in scatterplots in Figure 3.

Out of the 260 WQI calculated values at 15 monitoring stations, 23 values (8.2%) are between 0 and 25 (“excellent” water quality), 186 values (66.4%) are between 26 and 50 (“good”), 23 (8.2%) between 51 and 75 (“poor”), 9 (3.2%) between 76 and 100 (“very poor”), and 17 (6.0%) over 100 (“critical”).

It can be noticed that in Jiu and Olt tributaries WQI values are similar to those in the Danube at Gruia and Pristol, or lower, so these two rivers do not have a negative impact on Danube water quality. Values at Gruia and Pristol are also similar, so the Timok river, draining from Serbian territory between these stations, does not appear to have a negative impact on the Danube either.

The next two tributaries, Arges and Ialomita, are heavily polluted; however, water quality is similar upstream and downstream from their mouths, so their impact upon the Danube is also limited, because their flow is much smaller than the Danube flow.

In the Arges River, water quality was “very poor” or “critical” the whole period, with only three values under 100, in 1997, 2005 and 2014. This river receives insufficiently treated municipal sewerage waters from Bucharest, the capital city of Romania, through Dambovita River, Arges tributary. According to the National Institute for Statistics, Bucharest had a population of about 2.15 mil. inhabitants in January 2020 [59]. Until 2011 Bucharest wastewater treatment plant, Glina, only had mechanical step, and since 2011 it has advanced biological treatment for half of the incoming flow. Further upgrading is in progress.

Ialomita river is also more polluted than the Danube. The smallest WQI value was 51 in 2015, so all the values range from “poor” to “critical”. Ialomita also has the highest calculated value in the whole dataset, 247, in 2012. It is affected by sewerage from Slatina and Tandarei municipalities and has Prahova and Teleajen rivers as tributaries, bringing wastewater from industrial areas.

In Siret and Prut tributaries, water quality is worse than in the Danube, but better than in Arges and Ialomita. Their impact upon the Danube is also limited. Siret River had high WQI values related to high ammonium concentrations in 1996 and 1997, which could be related to sewerage pollution. The high value in 2005 corresponds to high total phosphorus values and high discharge, so it could be related to increased run-off during that year.

At Chilia, Sulina and Sf. Gheorghe, the three arms of the Danube forming the delta, all WQI values were “good” during the entire period, with only one “excellent” value at Sulina, in 2011.

Results show that WQI values are higher in some Romanian tributaries, particularly, Arges River, than in the Danube. In order to assess WQI trends in time, a statistical test is required.

### 3.2. WQI Trend Analysis

Trends were assessed using the Spearman rank correlation coefficients between calculated WQI values and the respective year. Positive coefficients indicate an increasing trend in time, negative coefficients indicate a decreasing trend.

The results of the WQI trend analysis for each monitoring station are presented in Table 6.

At 12 out of 15 monitoring stations WQI values have decreased in the analyzed period (1996–2017), which means that water quality has improved. For 3 Romanian tributaries the test result was not significant: Jiu, Arges, Ialomita.

Trend analysis shows that in the rivers with the highest WQIs (Arges and Ialomita) water quality has not improved, so this should be an alert for Romanian authorities managing these catchments to take stronger measures to reduce pollution.

### 3.3. Principal Component Analysis

WQIs were calculated based on 10 parameters and, in order to know which measures need to be taken to improve water quality, it is useful to know which parameters have the strongest influence on WQI values, and if there are correlations between them.

**Figure 4:**
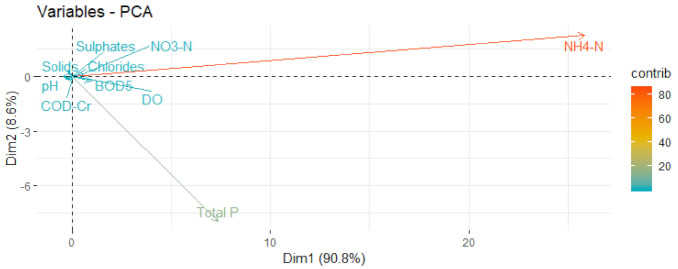
Principal Component Analysis on WQI sub-indices without scaling and centering for the period 1996–2017.

In the first step, principal component analysis (PCA) was applied on sub-indices (Qi*·*Wi/$\sum W_{i}$) without scaling and centering to find the parameters with highest variations after weighing. The result is shown in Figure 4, where arrows represent parameters, and the length of the arrow is proportional to the influence of the parameters on the WQI.

In Figure 4 it can be seen that ammonium dominates the first component (Dim1), which explains 90.8% of the variance of the dataset. The second important parameter is total phosphorus, dominating the second component (Dim2), which explains 8.6% of the variance. These two components explain 99.4% of the variance, so the dimensions of the dataset could be reduced from 10 to 2, without losing any important information.

This type of analysis reflects very well the weight of the parameters in the WQI, but it is difficult to see eventual correlations between them. For this reason, in the second phase, data were scaled and centered before PCA, so that relationships between parameters could be studied. The PCA coefficients, eigen values and variances for WQI sub-indices are presented in Table 7.

In Dim1 ammonium and sulfates have the highest coefficients, but TS, BOD_5_, chlorides, TP and nitrates also have important contributions. In Dim2 COD-Cr and dissolved oxygen are dominant. Dim1 explains 38.14% of the variance and Dim2 17.16%. The first five components explain together 81.03% of the variance, so the dataset could be reduced from 10 to 5 dimensions without losing important information.

**Figure 5:**
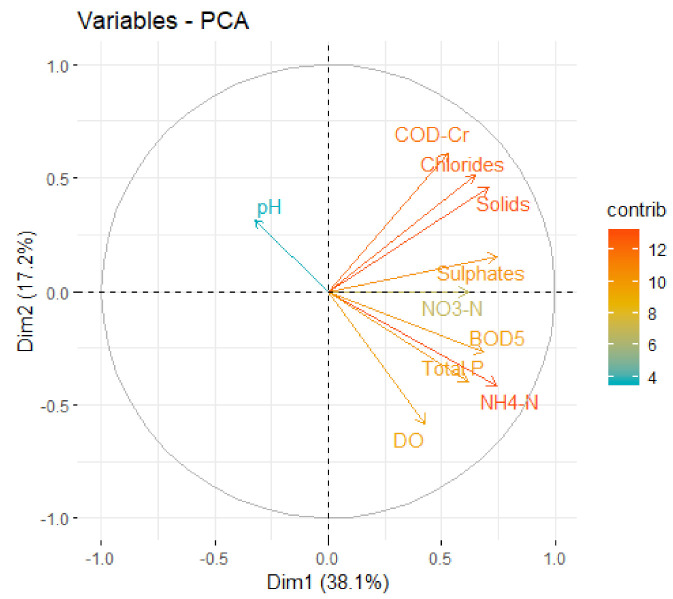
Graphical representation of first two principal components (Dim1 and Dim2) of WQI sub-indices PCA for the period 1996–2017.

Figure 5 is a graphical representation of parameters’ contributions to the first two principal components (Dim1 and Dim2), where arrows are parameters (variables), arrow lengths are proportional with parameters’ coefficients in the first two components, narrow angles between arrows indicate positive correlations, opposed arrows indicate negative correlations, and right angles indicate no correlation.

From Figure 5 it can be seen that there are strong positive correlations between chlorides and TS, ammonium and TP, and sulfates and nitrates. A negative correlation is between pH and DO, and no correlation between DO and solids. The negative correlation between pH and DO is induced by the calculation formula, because reference values (*V*_0_) are zero for all parameters, except for pH (7) and DO (14.6 mg O_2_/L), so DO sub-indices are inverted. When PCA is applied to annual means, there is a positive correlation between pH and DO [50].

The strong correlation between ammonium and TP, as well as the fact that ammonium has the highest variation in the dataset, are indications that the most important pollution factor is insufficiently treated municipal sewerage water.

### 3.4. Factor Analysis

**Figure 6:**
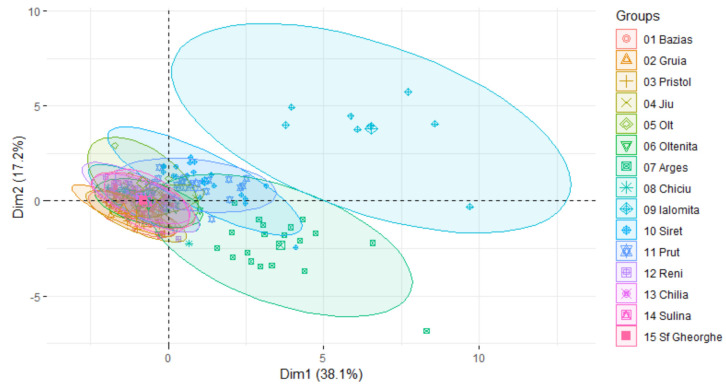
Graphical representation of WQI sub-indices PCA by location (1996–2017).

In order to identify which stations are more affected by pollution, PCA results were represented according to sampling locations in Figure 6, where parameters are represented by symbols, and locations by ellipses.

Water quality in the Ialomita River, the large ellipse in the upper right region, is most different from the stations on the Danube river, which are close to each other and to the axes. By ellipse position, in Ialomita COD-Cr, chlorides and TS have a strong influence on WQI.

The Arges River, the green ellipse in the lower right region, is also very different from the Danube, as it was already seen in WQI values, but in this case ammonium and total P are dominant. This river receives municipal wastewater from Bucharest through its tributary, Dambovita River, which is severely affected by sewerage pollution, as was demonstrated by Ionescu et al. [60].

Factor analysis further clarifies the differences between the studied locations. Apart from Ialomita and Arges, tributaries Siret and Prut, draining close to the Danube Delta, are also more polluted than the Danube, but much less than the first two.

Water quality in the first group of stations from Romania (Bazias, Pristol, and Gruia) is very similar to the last three stations, right before discharge into the Black Sea (Chilia, Sulina, and Sf. Gheorghe), as they are positioned close to each other, in the same area of the chart.

## 4. Discussion

The Danube has gone through significant alterations compared to the 19th century, due to human activities, with respect to water quality, sediment discharge, hydrology and morphology [15,32,39], impacting also species diversity in the river, as well as in the coastal region of the Black Sea where the Danube drains. The main sources of nutrients and organic pollution are insufficiently treated municipal sewerage water, industrial wastewater, manure from non-complying animal farms, and run-off from agricultural land where artificial fertilizers are not properly managed. In the 1980s, nutrients discharges led to eutrophication in the Black Sea, resulting in a large number of algal blooms, which caused oxygen depletion, affecting fish populations and their diversity [20]. This situation has changed during recent years in the Middle Danube, diatoms population indicating a shift towards an oligotrophic state [32].

This study calculated WQIs at 15 monitoring stations in the Lower Danube and tributaries in Romania for the period 1996–2017, by applying the weighted arithmetic method, using 10 parameters and limit values for water quality class II, as defined by Romanian legislation.

WQI results have shown that, during the studied period, pollution has decreased in the Lower Danube Basin at most of the analyzed locations. According to the applied method classification, water quality was “good” during most of the period at all stations in the Danube River. WQIs are lower for Romanian tributary Olt, which appears to be less polluted than the Danube. Jiu River WQI values are close to those for the Danube, while Siret and Prut were higher in the first part of the analyzed period but rank as “good” since 2007. A study carried out during the period 2013–2016 in Reni area has highlighted seasonal variations of WQIs, with better water quality during the winter season, but the calculated values ranked the water quality as ”poor”, mostly because some heavy metals were included in the index to account for industrial pollution [17].

Arges and Ialomita Rivers rank as “poor”, “very poor”, or “critical” during the entire period. Similar results were obtained for the Timok River, a Danube tributary from Serbia, using the Water Pollution Index for the period 1995–2009. In the case of Timok River, water quality got worse towards the end of the analysis period [61].

Trend analysis shows that water quality has improved significantly at 12 stations, while for Jiu, Arges and Ialomita Rivers there is no significant trend. The Third Joint Danube Survey, organized by ICPDR in 2013, found that, in general, Danube water quality has improved compared to previous surveys (in 2001 and 2007) with respect to nutrients, and that total nitrogen concentrations have decreased significantly, while total phosphorus only showed a slight decrease in the lower Danube [62].

PCA analysis of WQI sub-indices shows that the highest variance was given by ammonium and total phosphorus, indicating discharges of insufficiently treated municipal wastewater. Apart from nutrients concentrations, which allow an assessment based on chemical analysis, diatoms communities can offer valuable information on land use by reflecting environmental responses occurring in time to pressures such as urban agglomerations [16].

Urban sewerage is the main source of pollution of the Danube river and the most reliable safety indicator for drinking water is microbiological analysis. A thorough study on fecal pollution along the Danube during the Third Joint Danube Survey (2013) found that it was mostly of human origin and that contaminants were at critical levels after human agglomerations like Vienna, Budapest, Belgrade, and at strong or excessive levels at Kelheim, Russenski Lom, and Arges [18]. However, microbiological data are not available for long-term studies at the locations analyzed in the present study.

Factor analysis further confirmed that in Arges and Ialomita Rivers water quality is significantly lower than in the Danube, but values at Danube’s mouths (Chilia, Sulina, and Sf. Gheorghe) are similar to those at the first three stations in Romania (Bazias, Pristol, and Gruia), so the tributaries do not appear to have a strong impact on the Danube. The same conclusion has resulted from the Third Joint Danube Survey, which was carried out in 2013 [62].

Recent research has shown that mixtures of pollutants can pose environmental and public health threats even if each compound is below the allowed limit [29], so new methods are being developed for the ecological assessment of water bodies, including bioassays [63], diatoms analysis [16], antibiotic resistant genes [64] etc. In this respect, developing a water pollution index, that would aggregate several measured parameters, could provide a more accurate picture than screening of individual parameters [65].

The method applied in the present study could be of real use in developing such an index, because its flexibility allows it to be adapted according to available monitoring data. Source-pressure indices have already been used successfully in the development of an integrity model that can serve as a decision tool for authorities in the management of water bodies [2].

## 5. Conclusions

This study presents a complex methodology for assessing water quality, applied at 15 monitoring stations in the Lower Danube and Romanian tributaries for the period 1996–2017. At most locations WQIs decrease over time, indicating that water quality has improved, but some Romanian tributaries are more polluted than the Danube and still require efforts to improve wastewater treatment from urban agglomerations.

WQI values depend on the chosen calculation method even when applied on the same set of parameters, so it would not be appropriate to compare the results of the present analysis to values obtained in other works, but the improvement of individual parameters and reduced nutrients discharge are confirmed by several other studies, however for shorter periods, until 2009.

The applied methodology was useful for aggregating several parameters into one number to assess water quality and for identifying long-term trends at different locations, as well as for comparing locations in terms of pollution.

Sources of pollution of Ialomita and Arges Rivers require further attention, and efforts should be intensified to improve water quality in these rivers. The study can be useful to decision makers in defining intervention measures and prioritizing them, as well as for assessing the effects of the measures already taken and communicating them to the public.

## Figures and Tables

**Table 1:** Characteristics of monitoring locations [56].

Location Name	TNMN Location Code	Longitude	Latitude	River	Position along the River (km)	Catchment Area (km^2^)	Annual Mean Discharge m^3^/s
01 Bazias	RO1	21.38397	44.81610	Danube	1071.0	570,896	5515 *
02 Gruia	RO18	22.68355	44.27018	Danube	851.0	577,085	-
03 Pristol	RO2	22.67613	44.21418	Danube	834.0	580,100	5383 *
04 Jiu	RO19	23.84549	43.84188	Jiu	9.0	10,046	93 **
05 Olt	RO20	24.79680	43.74400	Olt	3.0	24,050	185 **
06 Oltenita	RO3	26.61905	44.05605	Danube	432.0	676,150	6107 *
07 Arges	RO9	26.59900	44.14500	Argeș	0.0	12,550	73 **
08 Chiciu	RO4	27.26771	44.12757	Danube	375.0	698,600	6113 *
09 Ialomita	RO21	27.66468	44.63477	Ialomița	24.0	10,309	48 **
10 Siret	RO10	28.00940	45.41474	Siret	0.0	42,890	235 **
11 Prut	RO11	28.20300	45.46900	Prut	0.0	27,480	110 *
12 Reni	RO5	28.23190	45.46324	Danube	132.0	805,700	6731 *
13 Chilia	RO6	29.55336	45.40635	Danube	18.0	817,000	3421 *
14 Sulina	RO7	29.52966	45.18338	Danube	0.0	817,000	1308 *
15 Sf Gheorghe	RO8	29.60945	44.88462	Danube	0.0	817,000	1761 *

* average based on TNMN monitoring data. ** [57].

**Table 2:** Number of values of selected parameters available at each location for the period 1996–2017.

	pH	DO	BOD_5_	COD-Cr	NH_4_N	NO_3_N	TP	TS	Cl^−^	SO_4_^−^
01 Bazias	332	319	356	298	379	379	348	378	317	314
02 Gruia	170	138	137	137	170	170	168	169	136	136
03 Pristol	384	351	417	332	457	457	426	452	350	347
04 Jiu	130	131	131	131	131	131	131	131	131	130
05 Olt	125	120	121	122	126	127	123	114	114	96
06 Oltenita	242	242	241	237	241	241	230	241	242	238
07 Arges	241	240	240	235	240	240	229	240	241	237
08 Chiciu	392	470	457	303	490	490	442	484	327	218
09 Ialomita	125	133	127	132	168	167	167	156	108	79
10 Siret	266	341	342	232	359	359	326	350	252	188
11 Prut	269	344	345	229	365	365	326	352	252	187
12 Reni	372	449	449	314	464	465	439	461	333	235
13 Chilia	252	252	249	226	258	258	233	258	249	208
14 Sulina	247	246	244	223	252	251	227	252	245	189
15 Sf Gheorghe	246	245	243	220	251	251	225	251	243	186

**Table 3:** Limit values (Si) and calculation of unit weights for selected parameters.

No.	Parameter	Unit	S_i_	1/S_i_	W_i_ = K/S_i_
1.	pH	-	7.5	0.1333	0.6172
2.	Dissolved Oxygen (DO)	mg O_2_/L	7	0.1429	0.6613
3.	Biochemical Oxygen Demand (BOD_5_)	mg O_2_/L	5	0.2000	0.9258
4.	Chemical Oxygen Demand (COD-Cr)	mg O_2_/L	25	0.0400	0.1852
5.	Ammonium Nitrogen (NH_4_^+^-N)	mg N/L	0.8	1.2500	5.7865
6.	Nitrates Nitrogen (NO_3_^−^-N)	mg N/L	3	0.3333	1.5431
7.	Total Phosphorus (TP)	mg P/L	0.4	2.5000	11.5730
8.	Total Suspended Solids (TS)	mg/L	750	0.0013	0.0062
9.	Chlorides (Cl^−^)	mg/L	50	0.0200	0.0926
10.	Sulfates (SO_4_^2+^)	mg/L	120	0.0083	0.0386
$\sum 1/S_{i}$ = 4.6292 $K=1/\left(\sum 1/S_{i}\right)$ = 0.216020491 $\sum W_{i}=21.4294$					

**Table 4:** Water quality rating by calculated water quality indices (WQI) values [44].

WQI	Water Quality
0–25	Excellent
26–50	Good
51–75	Poor
76–100	Very poor
Over 100	Critical

**Table 5:** Calculated WQI at each location for the period 1996–2017.

Location	WQI										
	1996	1997	1998	1999	2000	2001	2002	2003	2004	2005	2006
01 Bazias	30	38	40	29	49	41	32	30	35	41	29
02 Gruia	-	-	-	-	-	-	-	-	-	-	-
03 Pristol	34	37	38	32	45	34	31	29	30	53	31
04 Jiu	-	-	-	-	-	-	-	-	-	-	-
05 Olt	-	-	-	-	-	-	-	-	-	-	-
06 Oltenita	38	34	39	38	44	42	51	37	30	31	33
07 Arges	202	99	115	113	167	132	129	176	117	87	172
08 Chiciu	42	41	45	36	34	38	44	35	64	50	40
09 Ialomita	-	-	-	-	-	-	-	-	-	-	-
10 Siret	95	84	70	43	40	55	45	-	74	91	57
11 Prut	51	59	70	40	47	49	51	-	74	65	50
12 Reni	32	40	44	37	32	35	34	-	41	65	46
13 Chilia	34	40	47	37	26	37	33	-	43	39	40
14 Sulina	36	38	43	36	30	37	35	-	43	40	41
15 Sf Gheorghe	38	38	48	36	28	34	31	-	27	39	41
**Location**	**WQI**										
	**2007**	**2008**	**2009**	**2010**	**2011**	**2012**	**2013**	**2014**	**2015**	**2016**	**2017**
01 Bazias	34	58	54	28	27	32	26	24	26	-	22
02 Gruia	26	56	53	32	28	31	26	23	26	24	21
03 Pristol	28	60	57	33	30	32	25	22	25	-	21
04 Jiu	46	30	25	28	36	32	27	27	23	27	31
05 Olt	36	-	37	32	27	15	19	26	19	15	16
06 Oltenita	33	27	26	29	29	25	25	26	26	-	33
07 Arges	196	152	124	110	169	217	140	95	-	-	157
08 Chiciu	40	31	35	36	28	26	23	25	27	-	26
09 Ialomita	68	-	-	-	87	247	81	84	51	68	70
10 Siret	39	36	33	36	33	36	35	30	25	-	32
11 Prut	44	30	36	33	29	34	29	31	25	-	27
12 Reni	33	29	28	-	29	33	32	31	-	30	28
13 Chilia	33	31	29	28	26	31	31	31	-	-	29
14 Sulina	33	32	29	28	24	31	30	30	-	-	26
15 Sf Gheorghe	38	32	28	27	28	28	31	30	-	-	27

- data not available.

**Table 6:** Trend analysis of WQI values during the period 1996–2017.

Location	No. of Values	Spearman Correlation Coefficient ρ	*p* Value	Significant (*p* < 0.05)	Trend
01 Bazias	21	−0.5403	0.0126	Yes	↓decrease
02 Gruia *	11	−0.8273	0.0031	Yes	↓decrease
03 Pristol	21	−0.5623	0.0090	Yes	↓decrease
04 Jiu *	11	−0.3818	0.2484	No	
05 Olt *	10	−0.8061	0.0082	Yes	↓decrease
06 Oltenita	20	−0.7987	1.7525e^−05^	Yes	↓decrease
07 Arges	20	0.0842	0.7239	No	
08 Chiciu	21	−0.7429	0.0002	Yes	↓decrease
09 Ialomita *	7	−0.4524	0.2675	No	
10 Siret	19	−0.8752	0	Yes	↓decrease
11 Prut	20	−0.8195	6.7316e^−06^	Yes	↓decrease
12 Reni	19	−0.6123	0.0063	Yes	↓decrease
13 Chilia	19	−0.6105	0.0065	Yes	↓decrease
14 Sulina	19	−0.6667	0.0024	Yes	↓decrease
15 Sf Gheorghe	19	−0.5246	0.0228	Yes	↓decrease

* data available for the period 2007–2017.

**Table 7:** Results of principal component analysis (PCA) on centered and scaled sub-indices used in WQI calculation.

	Dim.1	Dim.2	Dim.3	Dim.4	Dim.5	Dim.6	Dim.7	Dim.8	Dim.9	Dim.10
NH_4_^+^-N	0.741	−0.416	0.060	−0.029	0.262	−0.200	0.159	−0.182	−0.160	−0.286
BOD_5_	0.686	−0.269	−0.181	0.221	−0.250	0.334	0.439	0.058	0.064	0.033
Chlorides	0.649	0.513	0.323	−0.114	0.067	0.065	0.088	−0.307	−0.176	0.239
COD-Cr	0.528	0.611	0.203	0.113	−0.259	−0.374	0.117	0.029	0.256	−0.083
DO	0.428	−0.584	0.005	−0.158	−0.590	−0.106	−0.255	−0.153	−0.033	0.050
NO_3_^−^-N	0.615	−0.003	−0.454	−0.525	0.121	−0.192	0.094	0.252	−0.017	0.130
pH	−0.325	0.315	−0.777	0.295	−0.111	−0.175	0.032	−0.198	−0.144	0.015
Sulfates	0.742	0.154	−0.340	−0.060	0.208	0.257	−0.287	−0.195	0.270	−0.072
Solids	0.705	0.460	0.012	0.140	−0.150	0.162	−0.237	0.283	−0.262	−0.134
Total P	0.617	−0.401	0.050	0.512	0.267	−0.201	−0.156	0.114	0.026	0.210
Eigenvalue	3.814	1.716	1.110	0.749	0.715	0.507	0.477	0.389	0.290	0.234
Explained variance %	38.14	17.16	11.10	7.49	7.15	5.07	4.77	3.89	2.90	2.34
Cumulative variance %	38.14	55.30	66.40	73.88	81.03	86.10	90.87	94.76	97.66	100.00

## Data Availability

Data used in this study were retrieved from the Danube River Basin Water Quality Database, available online: https://www.icpdr.org/wq-db/ (accessed on 2 January 2020) [52].

## Supplementary Materials

The following are available online at https://www.mdpi.com/1660-4601/18/4/1665/s1, Table S1: Annual Means, Table S2: WQI Sub-indices for PCA, Document S3: CORINE Land Cover Legend, Document S4: R-code.
