# Supplementary material for: Long-Term Trends in Water Quality Indices in the Lower Danube and Tributaries in Romania (1996–2017)
Source: Int J Environ Res Public Health. 2021 Feb 9;18(4):1665. doi: 10.3390/ijerph18041665 (PMC7916220; doi:10.3390/ijerph18041665)
Supplement: Supplementary file 1 [file ijerph-18-01665-s001.zip › Corine Land Cover Europe legend.docx]

| **Corine Land Cover Europe (2018)** |
| --- |


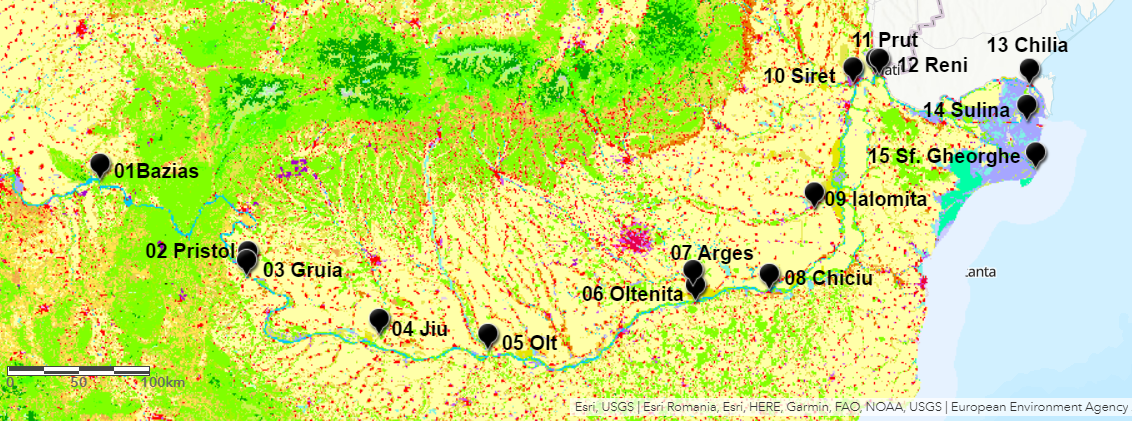


| Corine Land Cover 2018 raster |
| --- |

| 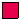 | \| Continuous urban fabric \| \| --- \| |
| --- | --- | --- |
| 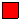 | \| Discontinuous urban fabric \| \| --- \| |
| 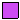 | \| Industrial or commercial units \| \| --- \| |
| 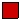 | \| Road and rail networks and associated land \| \| --- \| |
| 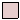 | \| Port areas \| \| --- \| |
| 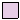 | \| Airports \| \| --- \| |
| 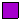 | \| Mineral extraction sites \| \| --- \| |
| 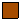 | \| Dump sites \| \| --- \| |
| 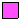 | \| Construction sites \| \| --- \| |
| 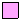 | \| Green urban areas \| \| --- \| |
| 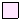 | \| Sport and leisure facilities \| \| --- \| |
| 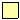 | \| Non-irrigated arable land \| \| --- \| |
| 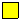 | \| Permanently irrigated land \| \| --- \| |
| 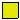 | \| Rice fields \| \| --- \| |
| 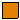 | \| Vineyards \| \| --- \| |
| 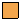 | \| Fruit trees and berry plantations \| \| --- \| |
| 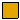 | \| Olive groves \| \| --- \| |
| 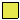 | \| Pastures \| \| --- \| |
| 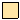 | \| Annual crops associated with permanent crops \| \| --- \| |
| 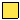 | \| Complex cultivation patterns \| \| --- \| |
| 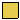 | \| Land principally occupied by agriculture, with significant areas of natural vegetation \| \| --- \| |
| 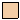 | \| Agro-forestry areas \| \| --- \| |
| 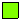 | \| Broad-leaved forest \| \| --- \| |
| 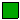 | \| Coniferous forest \| \| --- \| |
| 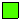 | \| Mixed forest \| \| --- \| |
| 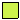 | \| Natural grasslands \| \| --- \| |
| 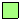 | \| Moors and heathland \| \| --- \| |
| 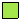 | \| Sclerophyllous vegetation \| \| --- \| |
| 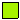 | \| Transitional woodland-shrub \| \| --- \| |
| 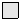 | \| Beaches, dunes, sands \| \| --- \| |
| 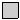 | \| Bare rocks \| \| --- \| |
| 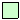 | \| Sparsely vegetated areas \| \| --- \| |
| 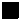 | \| Burnt areas \| \| --- \| |
| 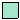 | \| Glaciers and perpetual snow \| \| --- \| |
| 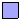 | \| Inland marshes \| \| --- \| |
| 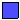 | \| Peat bogs \| \| --- \| |
| 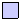 | \| Salt marshes \| \| --- \| |
| 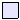 | \| Salines \| \| --- \| |
| 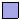 | \| Intertidal flats \| \| --- \| |
| 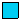 | \| Water courses \| \| --- \| |
| 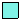 | \| Water bodies \| \| --- \| |
| 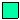 | \| Coastal lagoons \| \| --- \| |
| 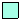 | \| Estuaries \| \| --- \| |
| 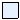 | \| Sea and ocean \| \| --- \| |
| 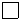 | \| NODATA \| \| --- \| |
