# Supplementary material for: Long-Term Trends in Water Quality Indices in the Lower Danube and Tributaries in Romania (1996–2017)
Source: Int J Environ Res Public Health. 2021 Feb 9;18(4):1665. doi: 10.3390/ijerph18041665 (PMC7916220; doi:10.3390/ijerph18041665)

> library(readxl)

> WQIPCA <- read_excel("C:/WQIPCA.xlsx")

> View(WQIPCA)

> library(tidyverse)

-- **Attaching packages** --------------------------------------- tidyverse 1.2.1 --

v ggplot2 3.1.0 v purrr 0.2.5

v tibble 2.1.3 v dplyr 0.8.3

v tidyr 1.0.2 v stringr 1.3.1

v readr 1.3.1 v forcats 0.3.0

-- **Conflicts** ------------------------------------------ tidyverse_conflicts() --

x dplyr::filter() masks stats::filter()

x dplyr::lag() masks stats::lag()

Warning messages:

1: package ‘tibble’ was built under R version 3.5.3

2: package ‘tidyr’ was built under R version 3.5.3

3: package ‘dplyr’ was built under R version 3.5.3

> library(psych)

Attaching package: ‘psych’

The following objects are masked from ‘package:ggplot2’:

%+%, alpha

> library(factoextra)

Welcome! Related Books: `Practical Guide To Cluster Analysis in R` at https://goo.gl/13EFCZ

> library(FactoMineR)

> theme_set(theme_bw())

> wqi.pca <- prcomp(WQIPCA[,c(3:12)])

> fviz_pca_var(wqi.pca, col.var = "contrib", gradient.cols = c("#00AFBB", "#E7B800", "#FC4E07"), repel = TRUE )


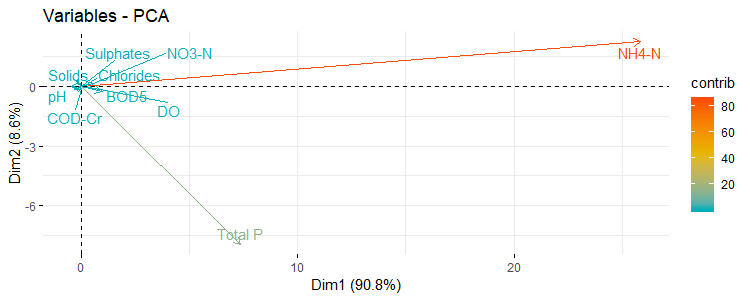


> wqi.pca <- prcomp(WQIPCA[,c(3:12)], center = TRUE,scale. = TRUE)

> fviz_pca_var(wqi.pca, col.var = "contrib", gradient.cols = c("#00AFBB", "#E7B800", "#FC4E07"), repel = TRUE )


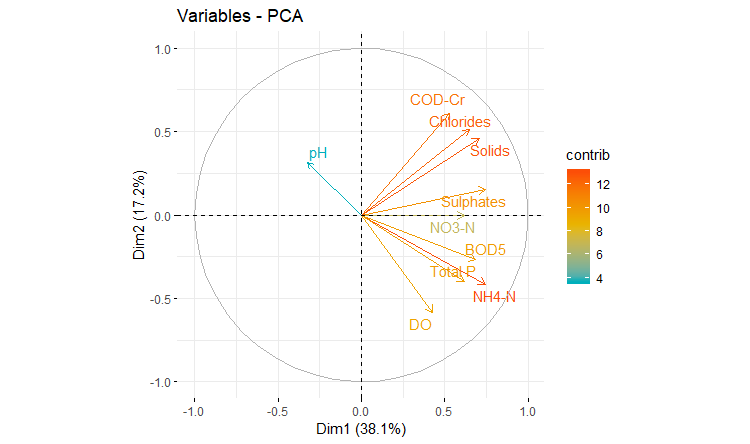


> wqi.pca <- prcomp(WQIPCA[,c(3:12)], center = TRUE,scale. = TRUE)

> fviz_eig(wqi.pca)


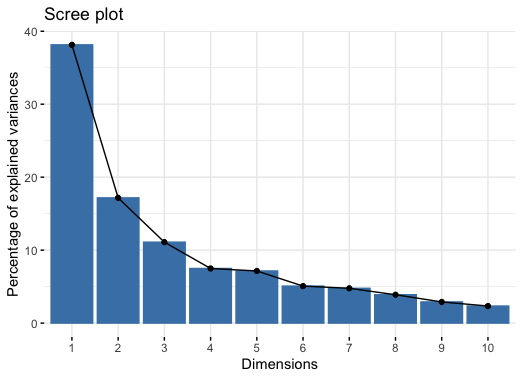


> eig.val <- get_eigenvalue(wqi.pca)

> eig.val

eigenvalue variance.percent cumulative.variance.percent

Dim.1 3.8137974 38.137974 38.13797

Dim.2 1.7164813 17.164813 55.30279

Dim.3 1.1095776 11.095776 66.39856

Dim.4 0.7486272 7.486272 73.88483

Dim.5 0.7145407 7.145407 81.03024

Dim.6 0.5073508 5.073508 86.10375

Dim.7 0.4769475 4.769475 90.87322

Dim.8 0.3886865 3.886865 94.76009

Dim.9 0.2901417 2.901417 97.66151

Dim.10 0.2338494 2.338494 100.00000

> res.var <- get_pca_var(wqi.pca)

> res.var$coord

Dim.1 Dim.2 Dim.3 Dim.4 Dim.5 Dim.6

N-NH4 0.7405221 -0.415826628 0.060285265 -0.02924566 0.26170119 -0.19996885

BOD5 0.6857644 -0.268973020 -0.180690200 0.22109671 -0.25029441 0.33427943

Chlorides 0.6487962 0.512532629 0.322681155 -0.11437727 0.06729384 0.06541083

CCO-Cr 0.5283332 0.610860021 0.203055397 0.11287260 -0.25878646 -0.37431540

DO 0.4277356 -0.583775250 0.004949193 -0.15809172 -0.58999488 -0.10647428

N-NO3 0.6147587 -0.003451833 -0.454080836 -0.52456939 0.12106198 -0.19166436

pH -0.3247473 0.314700995 -0.776938014 0.29538648 -0.11148355 -0.17545314

Sulphates 0.7416678 0.154155382 -0.339743078 -0.05996423 0.20762298 0.25685821

Solids 0.7053542 0.459591705 0.011741937 0.14016851 -0.15016996 0.16158511

Total P 0.6173626 -0.400690336 0.050297986 0.51225345 0.26658985 -0.20071929

Dim.7 Dim.8 Dim.9 Dim.10

N-NH4 0.15881187 -0.18151887 -0.16021607 -0.28620100

BOD5 0.43924681 0.05757087 0.06416818 0.03298910

Chlorides 0.08810596 -0.30738374 -0.17585969 0.23913878

CCO-Cr 0.11656956 0.02905390 0.25553384 -0.08324517

DO -0.25469329 -0.15263324 -0.03318898 0.05033335

N-NO3 0.09416479 0.25150052 -0.01685172 0.13001005

pH 0.03220232 -0.19844190 -0.14410344 0.01490918

Sulphates -0.28656691 -0.19526144 0.26957939 -0.07169240

Solids -0.23712424 0.28275807 -0.26193295 -0.13422417

Total P -0.15593674 0.11439558 0.02641106 0.20956785

> res.var$contrib

Dim.1 Dim.2 Dim.3 Dim.4 Dim.5 Dim.6 Dim.7

N-NH4 14.378662 1.007362e+01 0.327540249 0.1142503 9.5848303 7.8816359 5.2880473

BOD5 12.330828 4.214814e+00 2.942466464 6.5297865 8.7674911 22.0247483 40.4526170

Chlorides 11.037203 1.530397e+01 9.384033120 1.7474867 0.6337583 0.8433173 1.6275713

CCO-Cr 7.319109 2.173924e+01 3.715963184 1.7018117 9.3725143 27.6163982 2.8490476

DO 4.797258 1.985420e+01 0.002207553 3.3385099 48.7157630 2.2345036 13.6007990

N-NO3 9.909501 6.941614e-04 18.582693931 36.7570202 2.0511083 7.2405970 1.8591161

pH 2.765244 5.769752e+00 54.402025414 11.6550904 1.7393805 6.0675579 0.2174221

Sulphates 14.423187 1.384453e+00 10.402639807 0.4803070 6.0328683 13.0040478 17.2179515

Solids 13.045386 1.230567e+01 0.012425728 2.6244319 3.1560157 5.1462911 11.7891176

Total P 9.993623 9.353597e+00 0.228004550 35.0513055 9.9462702 7.9409028 5.0983106

Dim.8 Dim.9 Dim.10

N-NH4 8.4770369 8.8471229 35.02725538

BOD5 0.8527193 1.4191533 0.46537679

Chlorides 24.3087327 10.6591487 24.45478366

CCO-Cr 0.2171748 22.5053994 2.96334307

DO 5.9937520 0.3796450 1.08336659

N-NO3 16.2734008 0.0978765 7.22799213

pH 10.1313492 7.1571245 0.09505418

Sulphates 9.8091985 25.0474359 2.19791065

Solids 20.5698227 23.6466788 7.70415972

Total P 3.3668130 0.2404150 18.78075782

fviz_pca_ind(wqi.pca,

label = "none", # hide individual labels

habillage = WQIPCA$location, # color by groups

addEllipses = TRUE # Concentration ellipses

)


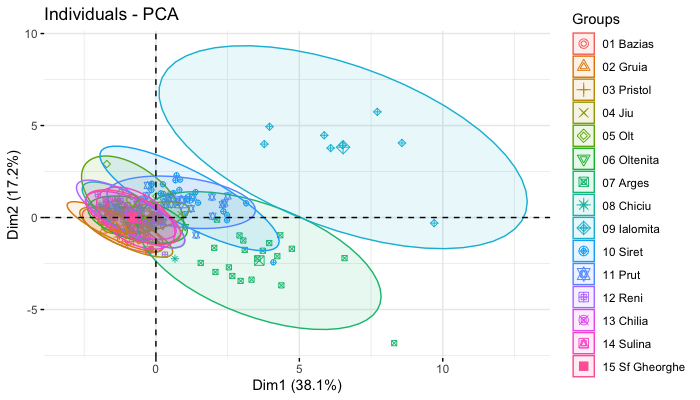


> medii <- WQIPCA[,-1,]

> res.famd <- FAMD(medii)

> fviz_famd_var(res.famd, "quali.var", repel = TRUE, col.var = "black")


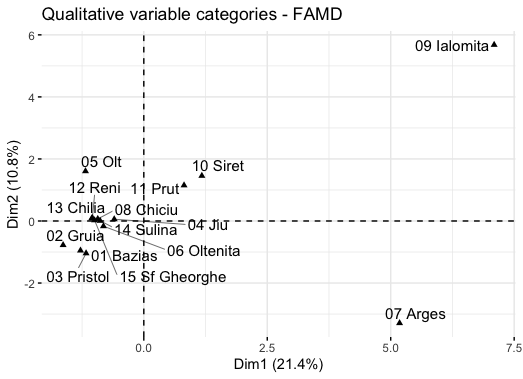


> wqi.coord <- res.var$coord

> write.csv(wqi.coord, file = "d:/wqicoord.csv")

> wqi.contrib <- res.var$contrib

> write.csv(wqi.contrib, file = "d:/wqicontrib.csv")

> summary(res.var)

Length Class Mode

coord 100 -none- numeric

cor 100 -none- numeric

cos2 100 -none- numeric

contrib 100 -none- numeric

> View(res.var)

> write.csv(eig.val, file = "d:/wqieig.csv")

ggplot(WQIPCA, aes(x = year, y = WQI)) + geom_point()+ facet_wrap(~location, scales = "free_y") + geom_hline(yintercept = 50, color = "blue")


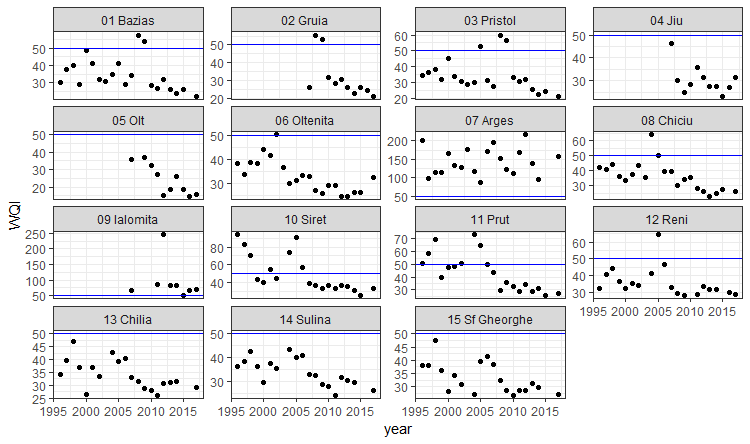


ggplot(means, aes(x = location, y = mean)) + geom_boxplot()+ facet_wrap(~determinand, scales = "free_y") + theme(axis.text.x=element_text(angle=90, vjust = 0.2, hjust=1))


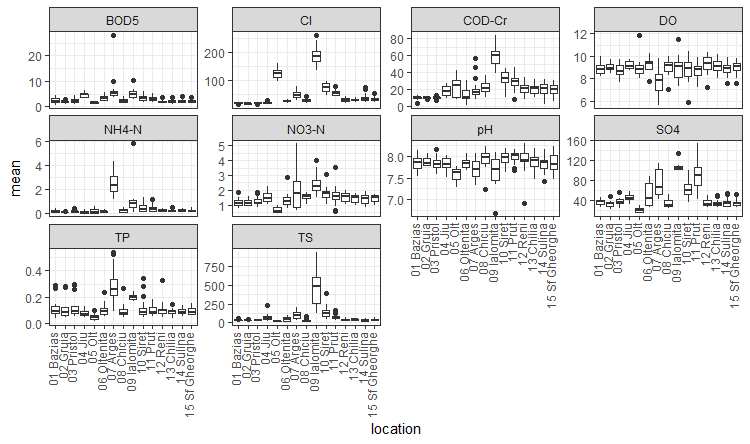

Supplement: Supplementary file 1 [file ijerph-18-01665-s001.zip › R code.docx]
